# Machine learning-based survival prediction in colorectal cancer combining clinical and biological features

**DOI:** 10.18632/oncotarget.28783

**Published:** 2025-12-15

**Authors:** Lucas M. Vieira, Natasha A.N. Jorge, João B. Sousa, João C. Setubal, Peter F. Stadler, Maria E.M.T. Walter

**Affiliations:** ^1^Department of Computer Science, University of Brasília, Campus Universitario Darcy Ribeiro, Prédio CIC/EST, Brasília, DF 71910-900, Brazil; ^2^Current Affiliation - Department of Pharmacology, School of Medicine, University of California San Diego, California, CA 92093, USA; ^3^Bioinformatics, Institute for Informatics, Leipzig University, Leipzig, Saxony 610101, Germany; ^4^Division of Coloproctology, Department of Surgery, University of Brasilia, Campus Universitario Darcy Ribeiro, Faculdade de Medicina, Brasília, DF 70910-900, Brazil; ^5^Institute of Chemistry, Department of Biochemistry, University of São Paulo, Av. Prof. Lineu Prestes, São Paulo, SP 05508-000, Brazil

**Keywords:** colorectal cancer, machine learning, feature selection, non-coding RNAs, genes

## Abstract

Colorectal cancer (CRC) is one of the most common and lethal types of cancer worldwide. Understanding both the biological and clinical aspects of the patient is essential to uncover the mechanism underlying the prognosis of the disease. However, most current approaches focus primarily on clinical or biological elements, which can limit their ability to capture the full complexity of the prognosis of CRC. This study aims to enhance understanding of the mechanisms of CRC by combining clinical and biological data from CRC patients with machine learning techniques (ML) to explore the importance of features and predict patient survival. First, we performed differential expression analysis and inspected patient survival curves to identify relevant biological features. Then, we applied ML techniques to understand the individual impact of each clinical and biological feature on patient survival. *E2F8*, *WDR77*, and *hsa-miR-495-3p* stood out as biological features, while pathological stage, age, new tumor event, lymph node count, and chemotherapy have shown themselves as interesting clinical features. Furthermore, our ML model achieved an accuracy of 89.58% to predict patient survival. The clinical and biological features proposed here in conjunction with ML can improve the interpretation of CRC mechanisms and predict patient survival.

## INTRODUCTION

Colorectal cancer (CRC) is one of the most common and lethal cancers in the world, accounting for around 10% of all cancer diagnoses in the world [1–4]. CRC occurs in the digestive tract, specifically in the colon, rectum, and rectosigmoid junction. The behavior and treatment of CRC can differ according to its anatomical site [5]. Although prognosis, prevention, and treatment have advanced due to the growing number of people diagnosed with CRC each year, a better understanding of the mechanisms of CRC development and progression continues to be crucial [3, 6–8].

Given the importance of mRNAs, miRNAs, and lncRNAs in cancer, recent studies also show the importance of their underlying interaction system in cancer progression, the so-called competing endogenous RNAs (ceRNAs) mechanism [9–12]. A ceRNA network can play an essential role in cancer development [13–15]. Therefore, exploring miRNAs, lncRNAs, mRNAs, and the ceRNA networks formed by them, together with clinical factors, could lead to a better understanding of the underlying mechanisms of CRC [16].

In this context, this article aims to present a method to predict patient survival in CRC by highlighting biological and clinical markers to characterize CRC behavior (and help in patient prognosis), taking into account the different anatomical sites: colon, rectum, and rectosigmoid junction. In more detail, it is common knowledge that interactions among proteins, miRNAs, and lncRNAs affect cancer since they can regulate suppressive and oncogenic functions in various types of cancer [14]. As is already known, understanding these mechanisms can help prevent tumor emergence and cancer development, as well as facilitate its identification. Although several studies present relationships between protein and ncRNAs with cancer [17–22], a few focus on predicting the prognosis of cancer patients using computational techniques with data from protein markers, miRNA, lncRNA, and clinical characteristics of patients [23–25], despite the fact that several databases present disease-related information [26–29] and specific information about cancer [30–34]. In recent years, research has been developed on different aspects of CRC, such as gene biomarkers for diagnosis [4, 35], prediction models for prognostics [36–38] and patient survival [39–42]. Recently, some interesting reviews about CRC and machine learning (ML) have been published [43, 44].

Other studies relate the importance of patient clinical aspects for cancer, e.g., the impact of race, age, and demographics on CRC emergence behavior [42, 45–47], but few [44, 48] explore the importance of these clinical aspects in combination with biological aspects to predict CRC prognosis and patient survival through machine learning.

## RESULTS

In this section, we first describe the data after the pre-processing execution, then the selected features, and finally, the results of the model construction.

### Data pre-processing

Using the method detailed in Section *Data pre-processing*, biological and clinical features were associated with a patient. For clinical features, it was noticed that, in many cases, information necessary to collect clinical metadata was missing. Of these features, the ones with the most missing values were race, ethnicity, weight, and height.

To address this problem, first, the clinical features were divided into two groups. Group (i) included age at initial pathological diagnosis, gender, number of lymph nodes, number of positive lymph nodes, chemotherapy, pathologic stage, vital status, and new tumor event. Group (ii) included race, ethnicity, weight, and height.

Then, considering these two groups of clinical features, data was grouped into three cases in the *missing features handler* step:

- Case 1. Filtered data with missing biological or Group (i) clinical features;
- Case 2. Filtered data with missing biological or Group (i) or Group (ii) clinical features; and
- Case 3. All data but replacing missing clinical features by using the most frequent value. In this case, missing values were filled using the most frequent value in the dataset. For example, if a patient’s race was missing and the most common race in the data was “white”, the patient was assigned to “white”. This approach was based on specialist recommendations, as features like race have fixed categories (e.g., “white” or “non-white”), and other imputation techniques, such as mean or median, could introduce non-existent values.

After filtering and transforming the data for each case, as described, the number of patients was: (i) for Case 1, 357 with colon cancer, 74 with rectum cancer, and 63 with rectosigmoid junction cancer; (ii) for Case 2, 177 with colon cancer, 27 with rectum cancer and 33 with rectosigmoid junction cancer; and (iii) for Case 3, 391 with colon cancer, 85 with rectum cancer, and 69 with rectosigmoid junction cancer. With all the features set up, prediction models were constructed for Cases 1, 2, and 3.

It is also important to note that, for the feature *new tumor event*, TCGA only indicates whether such an event occurred, without distinguishing between a metachronous tumor and a recurrence of the original tumor. Given that the incidence of metachronous colorectal cancer in sporadic populations is approximately 3–5% overall [49–51], and that only 20% of the selected cases were annotated as having a new tumor event, it is reasonable to assume that, on average, most of these events in our cohort represent recurrences rather than true metachronous tumors.

### Feature selection

**Figure 1:**
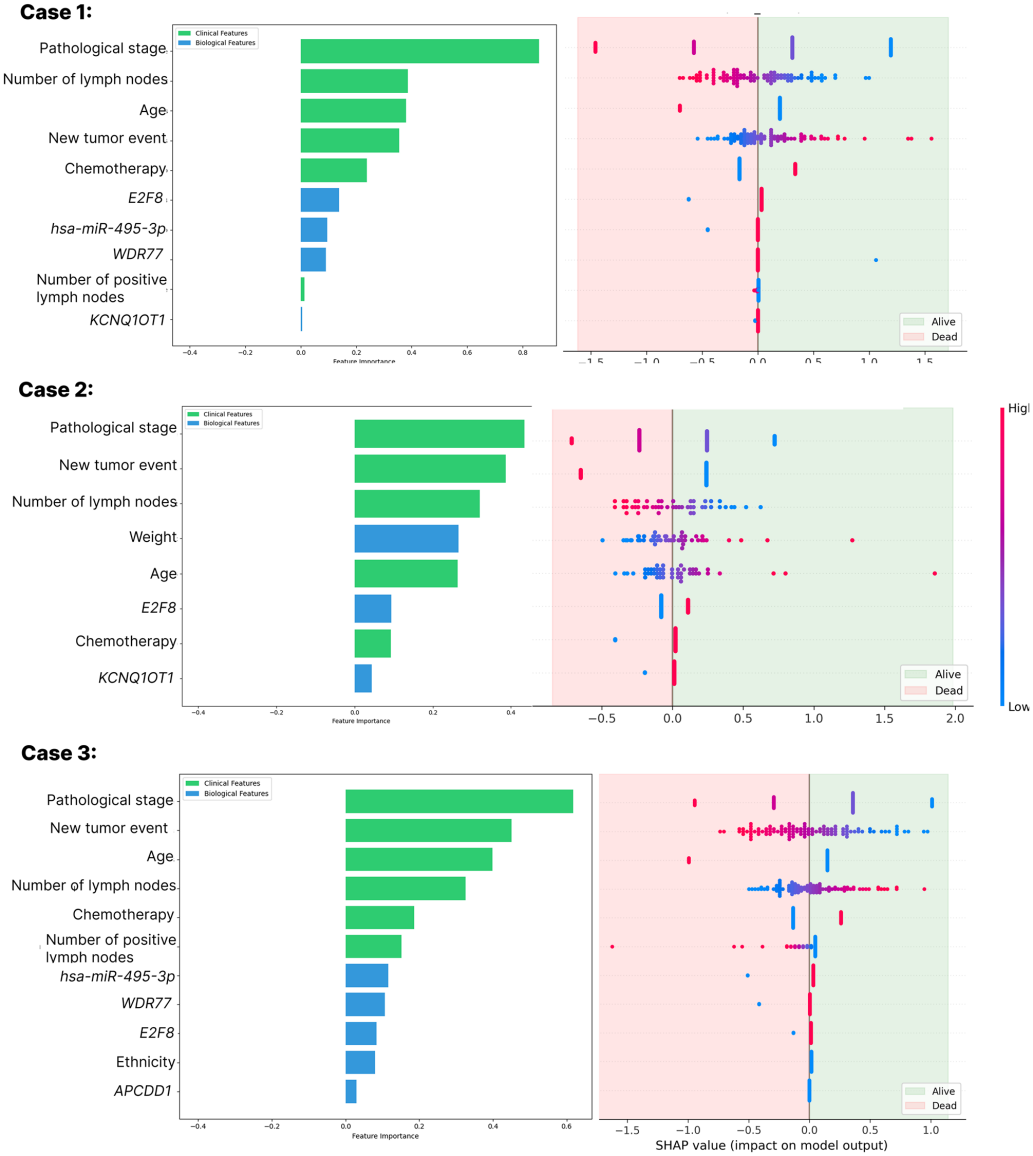
LASSO feature ranking and SHAP explanatory for Cases 1, 2, and 3 feature selection models. A positive SHA*P* value indicates a positive impact on prediction, leading the model to predict 1 (Patient survival). A negative value indicates an adverse effect, leading the model to predict 0 (Patient non-survival). The color of the SHAP data points shows the values as a heatmap where blue is the lowest value (e.g., 0) and red is the highest value (e.g., 1). For Cases 1 and 2, pathological stage and E2F8 expression are the most relevant clinical and biological features respectively. On the other hand, for group 3, pathological stage and hsa-miR-495-3p expression are the most relevant features.

During the feature selection phase, a grid search was applied to Least Absolute Shrinkage and Selection Operator (LASSO), in conjunction with a five cross-fold validation, to optimize its parameters and provide a more reliable process. Figure 1 - Case 1 shows the 10 features selected as important for Case 1. Among these features, six were clinical: pathological stage, number of lymph nodes, age, number of positive lymph nodes, new tumor event, and chemotherapy; and four were biological features: *E2F8*, *hsa-miR-495-3p*, *WDR77*, and *KCNQ1OT1*. When using Shapley Additive Explanations (SHAP) to improve understanding, it is possible to notice the impact of features such as pathological stage and age, which are directly related to a patient’s survival, as the greater its values are, the greater the chance of this patient not surviving.

Figure 1 - Case 2 shows the 7 features selected as important for Case 2. Among these features, five were clinical: pathological stage, new tumor event, number of lymph nodes, weight, age, and chemotherapy; and two were biological features: *E2F8* and *KCNQ1OT1*. In the analysis with SHAP, it is possible to notice the impact of features such as pathological stage, which is directly related to a patient’s survival, as the greater its values are, the greater the chance of this patient not surviving. Unlike Case 1, here, the age feature is inversely related to patient survival.

Figure 1 - Case 3 shows the 11 features selected as important for Case 3. Among these features, seven were clinical: pathological stage, new tumor event, age, number of lymph nodes, chemotherapy, number of positive lymph nodes, and ethnicity; and four were biological features: *hsa-miR-495-3p*, *WDR77*, *E2F8*, and APCDD1. With SHAP, it is possible to notice the impact of features such as pathological stage and age, which is directly related to patient survival, as the greater its values are, the greater the chance of this patient not surviving. Unlike Case 1 and similarly to Case 2, here, the *age* feature is inversely related to patient survival.

**Figure 2:**
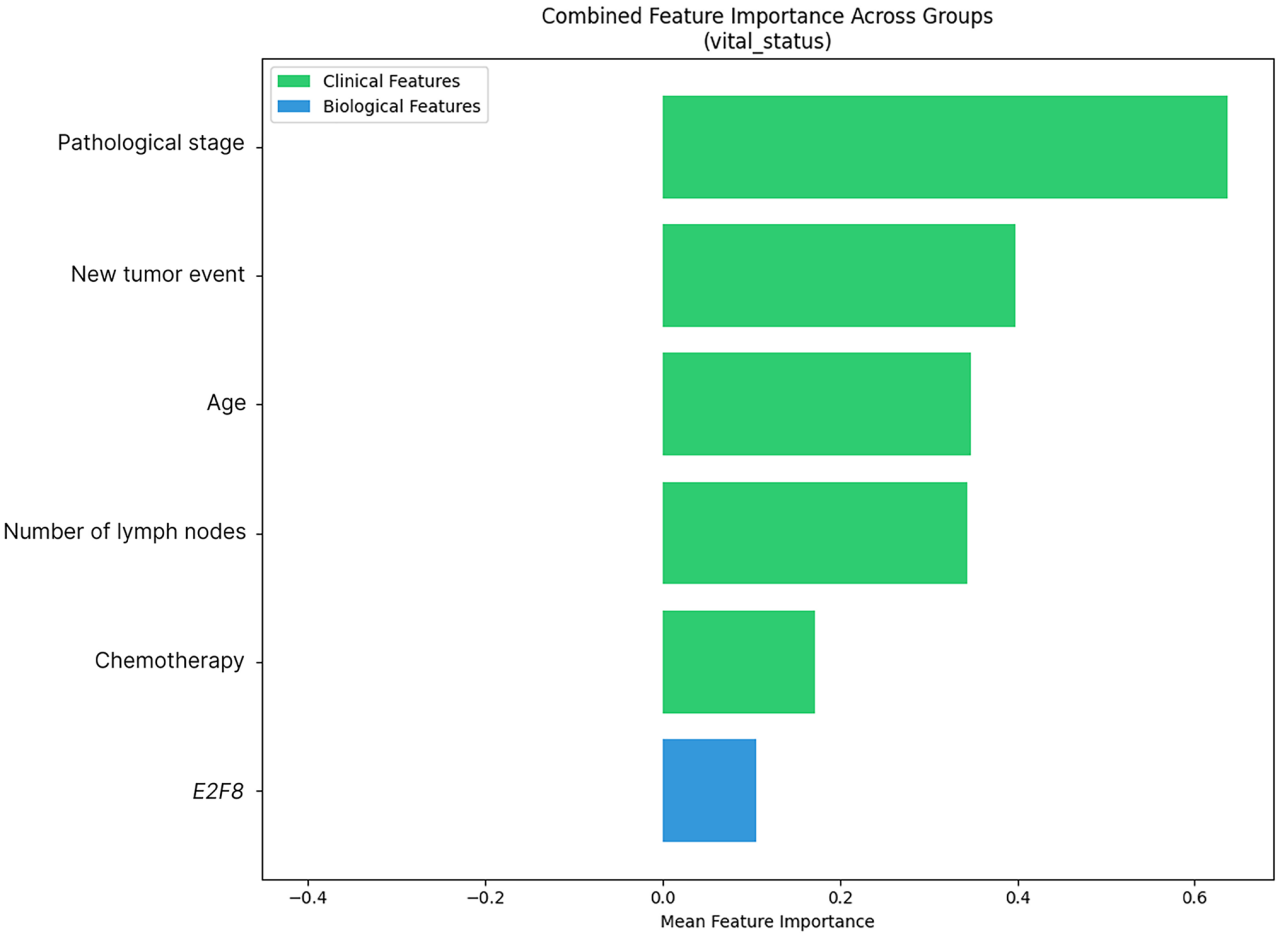
LASSO ranking for features relevant in groups 1, 2, and 3. The features are displayed with a mean importance value greater than 0 in all three groups. The meaning of importance scores of these selected features were computed and displayed to highlight their significance among the groups.

Figure 2 shows the standard features among the three groups. Compared to individual results, it is noticeable that the feature selection varies across the groups. Particularly for biological features, only the molecule *E2F8* was consistently ranked as important in all the models.

Finally, similar behavior is observed in Groups 1 and 3, including similarities in clinical and biological features, such as *WDR77* and *hsa-miR-495-3p*. This is different from Case 2, which may indicate that, when filtering with Case 2, the amount of remaining data may affect the prediction and selection of features.

### Model construction

After using LASSO in Phase 2, the features were used as input to construct the prediction model. During this phase, a grid search was applied in conjunction with a five cross-fold validation to optimize its parameters and prediction accuracy. Table 1 shows the performance evaluation of all the models constructed to predict patient survival using the selected features.

**Table 1:** Model performance for each group

Case	Model	Accuracy (%)	AUC (%)	Best parameters
1	LR	79.79%	81.77%	C: 0.1, weight: {0: 0.7, 1: 1.3}, solver: liblinear
	**SVM**	**86.87%**	**83.49%**	C: 0.1, weight: balanced, kernel: linear
	RandomForest	83.84%	79.37%	Weight: balanced, max depth: None, n: 200
	AdaBoost	80.81%	81.05%	Learning rate: 0.1, n: 50
	Stacking	81.82%	78.03%	ada n: 100, final estimator C: 1.0, rf n: 100
	Voting	80.81%	79.21%	ada n: 100, rf n: 100, svm C: 1
2	LR	70.83%	**82.05%**	C: 0.01, weight: {0: 0.7, 1: 1.3}, solver: liblinear
	SVM	77.08%	81.20%	C: 0.1, weight: balanced, kernel: linear
	RandomForest	85.42%	73.79%	Weight: balanced, max depth: None, n: 100
	**AdaBoost**	**89.58%**	76.50%	Learning rate: 0.1, n: 200
	Stacking	81.25%	77.49%	ada n: 100, final estimator C: 1.0, rf n: 100
	Voting	79.17%	74.07%	ada n: 100, rf n: 100, svm C: 1
3	LR	79.81%	79.02%	C: 0.01, weight: balanced, solver: lbfgs
	SVM	77.98%	**80.41%**	C: 0.1, weight: balanced, kernel: linear
	RandomForest	81.65%	76.25%	Weight: balanced, max depth: 20, n: 200
	AdaBoost	81.65%	76.44%	Learning rate: 0.1, n: 50
	Stacking	79.82%	78.84%	ada n: 100, final estimator C: 0.1, rf n: 100
	**Voting**	**82.57%**	78.27%	ada n: 100, rf n: 100, svm C: 1

*The highest accuracy and AUC are highlighted in bold for each group.*

The SVM model led to the best results in predicting patient survival for Case 1, achieving an accuracy of 86.87% on the test data, with 83.49% of AUC. The AB model led to the best accuracy for Case 2, achieving an accuracy of 89.58% and an AUC of 76.50%. The Voting model led to the best accuracy for Case 3, achieving an accuracy of 82.57% and an AUC of 78.27%. As shown, both the classical linear and the ensemble models led to a good performance in predicting patient survival using clinical and biological data. Still, the ensemble methods, in particular the Voting and Stacking approaches, display the overall best performance between groups.

Finally, the bootstrap analysis reveals varying levels of uncertainty among the adopted models and metrics. For Case 1, SVM shows the best performance with relatively narrow confidence intervals (±4.7% for accuracy). In Group 2, the AB model achieves the highest overall accuracy (89.58%), with wider confidence intervals (±7.0% for accuracy), indicating less certainty in its performance. In Group 3, the Voting classifier demonstrates the most consistent performance with narrow margins (±3.9% for accuracy). Statistical significance testing through bootstrap confidence intervals confirms that advanced ML models (particularly SVM, AB, and Voting classifiers) provide meaningful accuracy improvements over baseline logistic regression in all groups. However, AUC differences are generally not statistically significant due to substantial confidence interval overlaps across models.

## DISCUSSION

Feature selection and ML methods are widely used to understand large volumes of data better and to generate information. With the significant growth of biological data on CRC and the amount of information that can be extracted from these data to study CRC prognosis, the use of feature extraction techniques is of interest in improving ML methods [52, 53].

In this work, we used feature selection to identify biological and clinical features that are relevant to CRC patient survival. Also, ML models were created to predict patient survival, which can help doctors better understand key points in CRC prognosis. The proposed method combines biological and clinical features to predict patient survival, using as input data from patients from the United States, available in the TCGA database.

During the feature selection phase, using LASSO and SHAP, the results of this work suggest that the combination of biological and clinical features can help to understand and predict a patient’s prognosis. The biological feature with the most significant average impact in all cases was the *E2F8* gene, which was also identified by other studies as a potential CRC biomarker and is significantly associated with cell proliferation and indicates the CRC stage [54–56]. Although not present in Case 2, *WDR77* and *hsa-miR-495-3p* were also shown as relevant biological features for most of the groups, which was also identified by other studies [57, 58] as important in cancer or CRC development. Finally, age, pathological stage, chemotherapy, and lymph node count, which are clinical features previously identified as relevant in CRC development [59–63] were also highlighted as important in our study, even when combined with biological features.

Given these findings from the feature selection phase, our study supports the relevance of well-known clinical factors while also highlighting novel molecular factors. Furthermore, integrating clinical and molecular information, by combining established and newly identified features, improved the accuracy of prognostic prediction in colorectal cancer and contributed to refining prognosis across different stages of disease progression.

In the model construction phase, as expected with datasets storing only a few hundred cases and imbalanced labels, where non-survival cases are more prevalent, our bootstrapped confidence intervals indicated margins of approximately 5% to 10%. This, in comparison with the LR model used as a baseline, suggests that the ML models delivered meaningful improvements. Specifically, accuracy increased by 4.6% to 11.1%, while AUC showed only a low improvement of up to 4%. Although the results suggest a degree of similarity between LR and the more complex models for AUC, this can be attributed to the use of LASSO for feature selection, which can substantially enhance the performance of LR.

The ensemble methods and SVM consistently outperformed baseline LR for survival prediction. Using the ensemble AB model, we had the best accuracy of 89.58%, with an AUC of 76.50%. For patient survival prediction, AUC may be a more suitable metric due to the imbalance in the datasets since identifying high-risk patients is more critical than achieving high overall prediction accuracy. However, in this case, the ensemble methods demonstrated more excellent stability between both Accuracy and AUC. This suggests that combining simple models or even complex ones, such as SVM, RF, and AB, with a Voting or Stacking strategy can reduce overfitting since the metrics indicate more consistent performance. Focusing on stability across these metrics provides valuable insights into the robustness of the models, particularly in scenarios where no single metric fully captures performance.

Previous studies [24, 44, 52, 53] devised models to predict CRC-related outcomes through a variety of ML techniques. Alboaneen et al. [44] provided a systematic review of the application of ML for CRC detection and diagnosis, highlighting that ensemble methods such as RF can achieve strong performance. However, the results were different, depending on the dataset type and the pre-processing. Buk et al. [42] used clinical features and data from Brazilian CRC patients to predict survival, achieving an accuracy of 77% and AUC of 86% using RF. They also applied SHAP explanations to identify the most important features, among which were clinical stage and age. Achilonu et al. [24] created a pipeline using clinical features to predict CRC survival in South African patients. Specifically, their best model used an artificial neural network (ANN) and achieved an accuracy of 82.0%. Kang et al. [53] used LASSO to select biological and clinical features, such as age and sex, to predict lymph node metastasis in CRC. It obtained better results using LASSO than models without it, achieving the best AUC of 79.5%. In particular, Su et al. [52] also used TGCA-COAD data and applied LASSO to select biological features, along with SVM, RF, and DT, to predict colon cancer diagnosis and staging. For cancer diagnosis, they achieved high accuracy, reaching 98%. However, cancer staging proved to be more challenging, with a maximum accuracy of 91% and an AUC of 82%. Although these performance metrics might indicate overfitting, the techniques used have proven to be useful.

It is worth noting that the methods described in these studies, including our research, used different data and features as input and presented relatively good accuracy in predicting specific patient prognosis factors. ML methods use various clinical features to predict CRC prognosis targets [23, 24, 42, 44]. Achilonu et al. [24] and Gupta et al. [25] show that clinical features, such as age, gender, race, recurrence, chemotherapy, smoking, and alcohol consumption, can also lead to reasonable accuracy in predicting CRC prognosis factors. Our study identified some of the clinical features proposed in the cited articles as relevant, such as age, gender, race, recurrence, and chemotherapy. Although smoking and alcohol consumption have been shown to be appropriate in the cited works [23–25], these clinical features were not included in this study because their values were missing from the available TCGA data for most patients.

ML algorithms showed overall performance, particularly stacking, which displayed good overall results. Furthermore, the results of our work suggest that the combination of biological and clinical features can help predict patient prognosis. The biological feature with the most significant average impact in all cases was the *E2F8* gene, which was also identified by other studies as a potential CRC biomarker and significantly associated with cell proliferation and stages of CRC [54–56]. Although not present in Case 2, *WDR77* and *hsa-miR-495-3p* were also shown as relevant biological features for most of the groups, which was also identified by other studies [57, 58] as important in cancer or CRC development. Finally, age, pathological stage, chemotherapy, and lymph node count, which are clinical features previously identified as relevant in CRC development [59–63] were also highlighted as important in our study, even when combined with biological features.

Our study has some limitations. Initially, although several novel lncRNAs, mRNAs, and miRNAs with clinical significance for CRC were found, the study was developed using TCGA data and no further experimental validation was carried out. It is also essential to observe that TCGA consists of data collected exclusively from patients in the United States. In addition, some clinical features known to be relevant for CRC prognosis, such as smoking status and body mass index (BMI), were excluded from the training features due to missing information in most cases. For BMI, weight and height were available in some instances and showed trends supporting its prognostic importance, but the incomplete data limited their inclusion. The relatively small dataset (~545 patients), the use of only open-source data, and the imputation of missing categorical variables (e.g., race) also represent important constraints.

As acknowledged, these limitations underscore the value of dividing the data into groups for further exploration, which helped highlight the importance of collecting additional data, even though potential bias may have arisen from the imputation of missing categorical variables. CRC was treated as a single disease in our prediction instead of dividing it into its anatomical sites (colon, rectum, and rectosigmoid junction) to mitigate this limiting factor. This study also showed that even basic patient information, such as age and weight, when accurately recorded by doctors and stored on databases, can strongly contribute to improving CRC prognosis studies.

Finally, the development of this analysis with data collected from patients of other countries could give doctors a regional-specific view and a better understanding of CRC-specific characteristics for each anatomical site as potentially related to the region where patients live. Research on biological and clinical features in CRC is still under development. Further experimental studies and a more significant amount of CRC data are required to improve understanding of CRC prognosis.

In conclusion, this study proposed a model to understand biological and clinical features and predict CRC patient survival. For the best overall, the AB model resulted in an accuracy of 89.58% and an AUC of 76.50%. Furthermore, the results of this work suggest that the combination of biological and clinical features can help to predict patient prognosis. These results highlight evidence for prospective research of CRC associating *E2F8* as a potential prognostic biomarker and the importance of clinical features, e.g., age. The findings of this study can contribute to further studies on CRC using bioinformatic and ML techniques.

## MATERIALS AND METHODS

**Figure 3:**
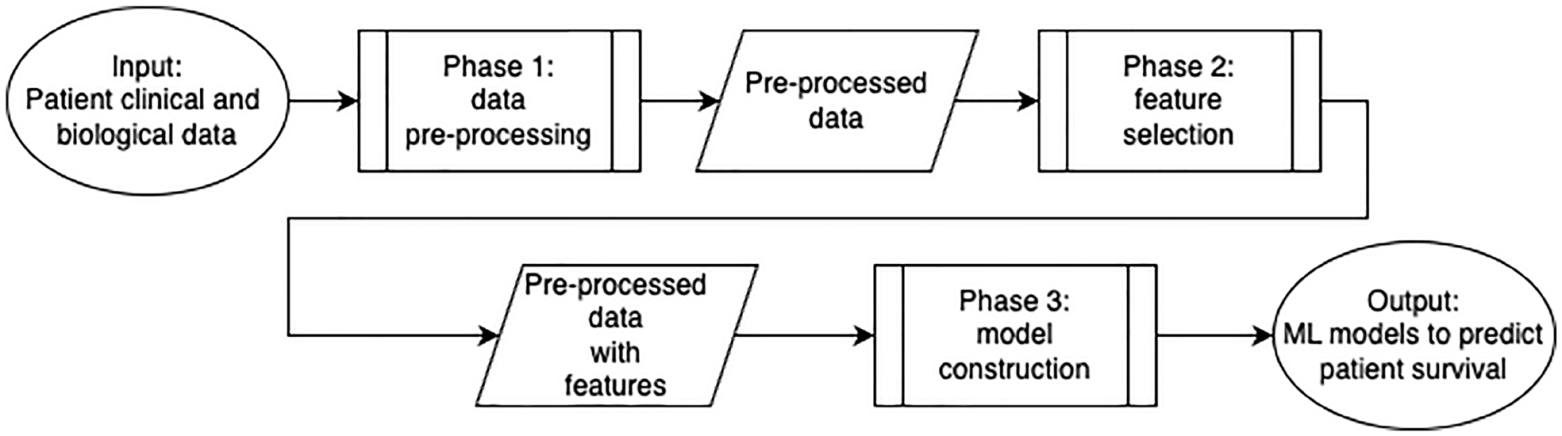
A method to predict CRC patient survival. The pipeline is divided into three main phases: (1) data pre-processing, in which the patient’s clinical and biological data is processed, (2) feature selection, in which clinical and biological features are ranked and then filtered; and (3) model construction, in which the prediction model is constructed and evaluated.

The model is designed to predict CRC patient survival. In practice, survival data record whether a patient survived after treatment until the last known medical appointment. The pipeline (Figure 3) is composed of three main phases: (1) data pre-processing; (2) feature selection, in which features are ranked and then filtered; and (3) model construction, in which the prediction model is constructed and evaluated. The pipeline was implemented in Python, using the scikit-learn [64] package for the ML algorithms implementation.

The input for the method was constructed with biological and clinical information downloaded from two databases, using the GDC interface (https://portal.gdc.cancer.gov/), TCGA rectal adenocarcinoma (TCGA-COAD)^1^; and TCGA rectal adenocarcinoma (TCGA-READ)^2^. Data were exclusively selected from adenocarcinoma, the most common type of CRC, and filtered to minimize variance by removing possible outlier cases. Therefore, the expression raw count data was collected from miRNA-seq and RNA-seq files at TCGA, with 541 primary tumors (TP) and 48 non-tumor tissues (NT), from 545 patients, whose 391 had colon cancer, 85 had rectum cancer and 69 had rectosigmoid junction cancer. The ages of the patients ranged from 31 to 90 years old, with an average age of 66. Of these, 185 patients (34%) received chemotherapy, 105 (19%) had a relapse, and 108 patients (20%) died. Details of the data can be seen on the project’s GitHub^3^.

### Data pre-processing

The data pre-processing phase consists of three steps: (1) *feature extraction*, which extracts the clinical and biological features from the input data; (2) *normalization*, which normalizes the clinical and biological data to numerical values; and (3) *missing features handler*, which construct the cases to be analyzed, taking into account the missing features of the data.

The feature extraction step uses the input data described previously to extract, process, and associate biological and clinical features for each patient. For biological features, as proposed by Vieira et al. [65], a customized script in R was developed. This custom script performs differential expression analysis to identify potential differentially expressed (DE) molecules. Based on these DE molecules, it constructs competing endogenous RNA (ceRNA) networks and subsequently conducts survival analysis using the DE molecules involved in these interactions. The output consists of candidate biological markers associated with patient survival, which are then used as biological features in downstream analyses.

The differential expression analysis was performed using GDCRNATools v1.6 [66], incorporating the limma package and voom normalization [67], with the thresholds *FDR* ≤ 0.05 and |*logFC*| ≥ 2. The ceRNA networks were constructed by predicting mRNA–miRNA–lncRNA interactions using the spongeScan algorithm [68] in conjunction with the StarBase v2.0 database [69], via a built-in function in GDCRNATools. The survival analysis was conducted using Cox proportional hazards (CoxPH) modeling and Kaplan–Meier (KM) estimation to calculate hazard ratios and generate survival curves, with (*p* < 0.05) considered statistically significant.

Finally, we select target biomarkers, in which: (i) the molecules are differentially expressed (DE); (ii) the biomarkers are presented in the CRC ceRNA networks; and (iii) the biomarkers affect patient survival. These criteria guaranteed the selection of molecules with a potential role in the CRC patient prognosis [65] and led to the compilation of a list of 19 molecules, as shown in Table 2.

**Table 2:** Candidate molecules to be used as biological features in the ML model to predict CRC patient survival

Molecule	Type	Potential roles in CRC
*ANKRD6*	Gene	Immune invasion [70]
*APCDD1*	Gene	CRC recurrence [71]
*DMD*	Gene	Lymph node metastasis [72]
*E2F8*	Gene	Cell proliferation [56]
*H19*	lncRNA	Cell migration and invasion [73]
*HECW2*	Gene	CRC progression [74]
*HOXD13*	Gene	CRC progression [75]
*KCNQ1OT1*	lncRNA	Chemo resistance [76]
*LINC00894*	lncRNA	Cell proliferation [77]
*NRG1*	Gene	Tumorigenesis [78]
*RANBP1*	Gene	CRC progression [79]
*SNHG16*	lncRNA	Cell growth [80]
*TMEM198*	Gene	CRC prognosis [65]
*UST*	Gene	CRC prognosis [65]
*WDR77*	Gene	Cell proliferation [57]
*hsa-miR-1271-5p*	miRNA	Cell proliferation [81]
*hsa-miR-130a-3p*	miRNA	Cell proliferation [82]
*hsa-miR-130b-3p*	miRNA	Cell growth [83]
*hsa-miR-495-3p*	miRNA	Cell proliferation [58]

To parameterize these molecules as biological features, we developed a customized Python script using the expression data calculated in the previous script as input. This script utilized two key elements from the input: the molecule’s average voom-normalized expression, and its normalized counts for each patient. Using this information, the script determined whether each molecule was overexpressed in a patient by checking if its normalized expression count was higher than the normalized average expression *E_patient_ > AveExpr*.

To identify clinical characteristics, we proceeded as follows. First, the raw clinical metadata available at TCGA was analyzed. These clinical features were divided into 9 groups - clinical, demographic, diagnosis, exposure, family history, follow-up, molecular test, pathological details, and treatment. A doctor specialist in CRC assisted in the process of manually choosing the most relevant characteristics from the available data. The following features were selected: age at initial pathological diagnosis, ethnicity, gender, race, vital status, number of positive lymph nodes, number of lymph nodes, pathological stage, weight, height, chemotherapy, new tumor event, and vital status.

To normalize and prepare the data to be used in the prediction models, in the *normalization* step, the clinical and biological features were transformed into numerical values, as shown in Table 3. These numerical values were later used in the charts to show the importance of the features.

**Table 3:** List of numerical values used in the feature vector

Feature	Associated values
Age	Numerical value = age of the patient
Chemotherapy	1 = received chemo; 0 = did not receive chemo
Ethnicity	1 = Latino; 0 = non Latino
Gender	0 = female; 1 = male
Height	Numerical value = height of the patient
Race	1 = non-white; 0 = white
Pathological stage	Stage IV = 3; stage III = 2; stage II = 1; stage I = 0
Vital status	1 = survival; 0 = non-survival
Number of positive lymph nodes	Numerical value = number of lymph nodes
Number of lymph nodes	Numerical value = number of positive lymph nodes
Weight	Numerical value = weight of the patient
New tumor event	1 = new tumor; 0 = no new tumor
*ANKRD6*	1 = overexpressed; 0 = not overexpressed
*APCDD1*	1 = overexpressed; 0 = not overexpressed
*DMD*	1 = overexpressed; 0 = not overexpressed
*E2F8*	1 = overexpressed; 0 = not overexpressed
*H19*	1 = overexpressed; 0 = not overexpressed
*HECW2*	1 = overexpressed; 0 = not overexpressed
*HOXD13*	1 = overexpressed; 0 = not overexpressed
*KCNQ1OT1*	1 = overexpressed; 0 = not overexpressed
*LINC00894*	1 = overexpressed; 0 = not overexpressed
*NRG1*	1 = overexpressed; 0 = not overexpressed
*RANBP1*	1 = overexpressed; 0 = not overexpressed
*SNHG16*	1 = overexpressed; 0 = not overexpressed
*TMEM198*	1 = overexpressed; 0 = not overexpressed
*UST*	1 = overexpressed; 0 = not overexpressed
*WDR77*	1 = overexpressed; 0 = not overexpressed
*hsa-miR-1271-5p*	1 = overexpressed; 0 = not overexpressed
*hsa-miR-130a-3p*	1 = overexpressed; 0 = not overexpressed
*hsa-miR-130b-3p*	1 = overexpressed; 0 = not overexpressed
*hsa-miR-495-3p*	1 = overexpressed; 0 = not overexpressed

Finally, in the *missing features handler* step, the data points with any missing feature were removed or had their values replaced by the feature median value. Experiments were developed for both cases and are detailed in Section *Results*.

### Feature selection

The feature selection phase consisted of two steps using Least Absolute Shrinkage and Selection Operator (LASSO): (1) *feature ranking*, in which the grid search is used to select the best training parameters and to rank each biological and clinical feature by importance in the final prediction, and (2) *feature selection*, in which the most relevant features are selected.

After using LASSO for feature selection, Shapley Additive Explanations (SHAP) was used to assess the impact of each selected feature on the prediction. This was done to provide an intuitive understanding of how each predictor contributes to classifying the patient’s vital status.

### Model construction

The model construction phase consisted of five steps: (1) *data split*, which divides the pre-processed data into training and testing data in an 80% and 20% ratio; (2) *feature selection*, in which the features obtained at Phase 2 (feature selection) are selected; (3) *parameter optimization*, where grid search and cross-validation were used to optimize the ML hyperparameters; (4) *ML classifiers construction*, in which ML models using Logistic Regression (LR), Support Vector Machine (SVM), Random Forest (RF), Adaptative Boosting (AB), Stacking (SE), and Voting (VE) are built; and (5) *performance evaluation*, in which the ML classifiers are evaluated and compared.

In the *ML classifiers construction* step, the six classifiers (LR, SVM, RF, AB, SE, and VE) were used since each behaves differently based on the pattern of the input data. Therefore, the goal was to explore these classifiers to find the best option to predict the expected outcome. In particular, the SE and VE classifiers used the combination of SVM, RF, and AB, adopting an ensemble voting and stacking strategy to verify whether using multiple classifiers together could improve the outcome. Then, in the *performance evaluation* step, the performance of each ML model was evaluated, taking the testing data as input and comparing the models through several metrics, such as AUC, accuracy, precision, and recall.

Finally, to validate the performance of the proposed model, the LR model was constructed as a baseline for comparative analysis due to its interpretability and simplicity. Furthermore, to address potential prediction errors arising from the limited number of data, stratified bootstrapping was adopted to preserve class balance, using 1,000 iterations and a confidence level of 95% to estimate prediction confidence intervals.

## Footnotes

^1^ https://portal.gdc.cancer.gov/projects/TCGA-COAD

^2^ https://portal.gdc.cancer.gov/projects/TCGA-READ

^3^ https://github.com/lmacielvieira/crc-bio-cli-ml

## Abbreviations

AB: Adaptative Boosting
AUC: Area Under the Curve
ceRNAs: competing endogenous RNAs
CRC: Colorectal cancer
DE: differentially expressed
LASSO: Least Absolute Shrinkage and Selection Operator
LR: Logistic Regression
ML: Machine learning
RF: Random Forest.
SE: Stacking ensemble
SHAP: Shapley Additive Explanations
SVM: Support Vector Machine
VE: Voting ensemble
